# The Mechanism of PEDV-Carrying CD3^+^ T Cells Migrate into the Intestinal Mucosa of Neonatal Piglets

**DOI:** 10.3390/v13030469

**Published:** 2021-03-12

**Authors:** Chen Yuan, Yuchen Li, En Zhang, Yuxin Jin, Qian Yang

**Affiliations:** MOE Joint International Research Laboratory of Animal Health and Food Safety, College of Veterinary Medicine, Nanjing Agricultural University, Weigang 1, Nanjing 210095, Jiangsu, China; yuanchen060624@163.com (C.Y.); yuchengli0016@126.com (Y.L.); MrJohn1994@163.com (E.Z.); jyx843729845@163.com (Y.J.)

**Keywords:** PEDV, CD3^+^ T cells, neonatal piglets, intranasal inoculation

## Abstract

Porcine epidemic diarrhea virus (PEDV) can cause intestinal infection in neonatal piglets through the nasal cavity. A process in which CD3^+^ T cells carry PEDV plays a key role. However, the modes through which PEDV bridles CD3^+^ T cells as a vehicle for migration to the intestinal epithelium have not been clarified. In this study, we first demonstrated that PEDV could survive in blood-derived CD3^+^ T cells for several hours, depending on the multiplicity of infection. In addition, PEDV preferentially survived in CD4^+^ T cells over CD8^+^ T cells. Moreover, viral transmission was mediated by cell-to-cell contact between mesenteric lymph-node-derived CD3^+^ T cells, but did not occur in blood-derived CD3^+^ T cells. Following an increase in gut-homing integrin α4β7, blood-derived CD3^+^ T cells carrying PEDV migrated to the intestines via blood circulation and transferred the virus to intestinal epithelial cells through cell-to-cell contact in neonatal piglets. Our findings have significant implications for understanding PEDV pathogenesis in neonatal piglets, which is essential for developing innovative therapies to prevent PEDV infection.

## 1. Introduction

Porcine epidemic diarrhea virus (PEDV) is the causative agent of porcine epidemic diarrhea (PED), an acute and highly contagious enteric viral disease [1,2]. Although PED can occur in swine of any age, neonatal piglets are the most severely affected [3,4]. The condition is characterized by watery diarrhea, dehydration, and vomiting, and outbreaks have occurred on swine farms in Asia, Europe, and America [4,5,6]. The fecal–oral route is believed to be the primary mode of PEDV transmission. Recently, an alternative pathway of enteric PEDV dissemination from the nasal cavity to the intestinal mucosa in swine was reported [7], clarifying that dendritic cells (DCs) distributed beneath the nasal mucosa of piglets can capture PEDV by forming transepithelial dendrites (TEDs). PEDV-carrying DCs can transfer the virus to CD3^+^ T cells via virologic synapses. Finally, virus-loaded CD3^+^ T cells reach the intestines via blood circulation, leading to intestinal infection through cell-to-cell contact [7]. A recent study reported that subepithelial CD3^+^ T cells mediated PEDV infection through the nasal cavity in neonatal piglets [8]. However, this report did not reveal a detailed mechanism by which CD3^+^ T cells transmit PEDV to the intestines via blood circulation.

Viruses can bridle lymphocytes to reach the target organs and cause infection [9,10]. Lymphocytes carrying viruses are crucial for efficient viral spread among permissive target cells and systemic spread during infection. Moreover, the formation of virological synapses during stable contact between infected and uninfected lymphocytes substantially increases the efficiency of viral transfer, thereby increasing transmission [11]. Reportedly, viruses are preferentially transmitted between immune cells, which is considerably more efficient than transmission involving free viral particles [12]. Therefore, virus transmission between immune cells plays an important role in viral infection [13,14]. PEDV can be transmitted between immune cells through formation of viral synapses between DCs and CD3^+^ T cells. However, it is uncertain whether PEDV can be transmitted within CD3^+^ T cells.

The expression of C-C chemokine receptor 9 (CCR9) and integrin α4β7 in T cells promotes their trafficking to the gut via interactions with C-C chemokine ligand (CCL25) and mucosal addressin cell adhesion molecule 1 (MAdCAM-1), both of which are highly expressed in mucosal cells of the small intestine. Neonatal lymphocyte maturation and homing receptors differ from those of adult lymphocytes; neonatal lymphocytes predominantly express the gut-homing receptor α4β7 [15,16]. Furthermore, numerous studies have suggested that human immunodeficiency virus type-1(HIV-1) can not only increase the expression of integrin α4β7, but can also incorporate integrin α4β7 in lymphocytes to promote lymphocyte-carrying virus migration to gut-associated lymphoid tissue [17,18,19]. However, it is not clear whether the expression of CCR9 and integrin α4β7 is elevated in CD3^+^ T cells that carry PEDV.

In the present study, we found that PEDV could hijack blood-derived CD3^+^ T cells in vitro and in vivo. Subsequently, CD3^+^ T cells carrying PEDV can be transmitted between MLN-CD3^+^ T cells, but not blood-derived CD3^+^ T cells. Furthermore, integrin α4β7 was expressed at high levels in PEDV-infected blood-derived CD3^+^ T cells, promoting their trafficking to the gut via interactions with MAdCAM-1. Finally, blood-derived CD3^+^ T cells carrying PEDV reach the intestines via blood circulation, where they cause intestinal infections through cell-to-cell transmission in neonatal piglets. These results have significant implications for understanding PEDV pathogenesis in neonatal piglets, which is essential for developing innovative therapies to prevent PEDV infection.

## 2. Material and Methods

### 2.1. Reagents and Cell Lines

Integrin α4β7 and Anti-CCR9 were purchased from Novus (Fort Collins, CO, USA) and Abcam (Cambridge, London, UK) respectively. Alexa FluorR 647 Mouse Anti-Pig CD3 (8BB23-8E6-8C8), PE Mouse Anti-Pig CD8 (76-2-11), and FITC Mouse anti-Pig CD4 (74-12-4) were purchased from BD Biosciences (USA). The anti-PEDV N protein mAb was purchased from Medgene Labs (Vermillion, SD, USA). Carboxyfluorescein succinimidyl amino ester (CFSE) was purchased from Invitrogen (Waltham, MA, USA). Phytohemagglutinin (PHA) and Red Fluorescent Cell Linker Kits (PKH26) were purchased from Sigma-Aldrich (St. Louis, MO, USA). Anti-APC (130-097-143) and MiniMACS Starting Kits were all purchased from Miltenyi Biotec (Bergisch-Gladbach, Germany). C-C chemokine ligand 25 was purchased from Peprotech (Rocky Hill, NJ, USA). Vero E6 cells were kindly provided by the Veterinary Medicine Research Center of the Da Bei Nong Group. The cell line was regularly tested for mycoplasma contamination according to the specifications of the Mycoplasma Stain Assay Kit.

### 2.2. Animal

Conventional Duroc × Landrace × Yorkshire neonatal piglets (1-day-old) were obtained from a swine herd at the Jiangsu Academy of Agricultural Science (JAAS). The neonatal piglets were born via natural farrow and were fed with synthetic milk. The swine herd was seronegative for antibodies against PEDV, porcine reproductive and respiratory syndrome, porcine Respiratory Coronavirus, transmissible gastroenteritis virus, and porcine circovirus type 2. Each experimental group of neonatal piglets was housed in a separate room, with constant humidity and temperature and a 12 h light/dark cycle. All procedures and experiments performed on the animals were approved by the Institutional Animal Care and Use Committee of Nanjing Agricultural University and followed the National Institutes of Health guidelines for the performance of animal experiments (Permit Number: SYXK2017-0027, 20 June 2017).

### 2.3. Virus

The wild-type PEDV strain Zhejiang08 was preserved by our laboratory, which was clustered with the emerging virulent strain [7].

### 2.4. Vascular Fistula in Neonatal Piglets

Neonatal piglets were intranasally inoculated with 1 mL PEDV (10^6^ plaque-forming units per mL). Then, neonatal piglets were anaesthetized and isoflurane was used for maintenance anesthesia. Following standard surgical cleansing, a skin incision was made. Fascial and muscle planes were carefully dissected out with attention to hemostasis. The precaval vein was identified and dissected out. A vascular fistula was then made in precaval vein and the skin was anastomosed to the precaval vein and artery using polypropylene sutures. Special attention was paid to ensure that there was no kinking or torsion of the completed fistula. Blood was collected in anticoagulant tubes at different times for fluorescence-activated cell sorting (FACS).

### 2.5. Generation of Lymphocytes

Porcine PBMCs (Peripheral blood mononuclear cells) were isolated from the blood of neonatal piglets by density centrifugation using a porcine peripheral blood lymphocyte separation kit (Solarbio, Beijing, China). The method used to isolate lymphocytes from mesenteric lymph nodes (MLN) is as follows. The neonatal piglets were euthanized using pentobarbital sodium. To determine complete anesthetization of the piglets before opening the abdomen, their palpebral and withdrawal reflexes were checked. After removal of residual mesenteric fat tissue, the lymph node was then cut into 0.5 cm pieces. The pieces were incubated in 20 mL of 10 mM EDTA in HBSS for 20 min at 4 °C, then centrifuged, discarded, and placed in digestion solution containing 4% fetal bovine serum, 2 mg/mL each of collagenase D (Roche, Switzerland) and DNase I (Sigma, USA), and 100 U/mL dispase (Thermo Fisher Scientific, Waltham, MA, USA) at 37 °C for 20 min with slow rotation. The cells were obtained by density gradient centrifugation. Then, CD3^+^ T cells were sorted from PBMCs or MLNs by anti-APC MicroBeads and were activated by PHA and IL-2 for three days for subsequent experiments. All subsequent experiments were performed with blood-derived CD3^+^ T cells activated by PHA and IL-2, unless stated differently.

### 2.6. Cell-to-Cell Contact

Blood-derived CD3^+^ T cells, untreated or treated with PEDV (MOI = 0.1), were labeled with PKH26 according to the manufacturer’s instructions. The blood-derived CD3^+^ T cells (5 × 10^6^ cells/mL) were labeled with 10 nM CFSE for 10 min at 37 °C. The PEDV-pulsed, blood-derived CD3^+^ T cells labeled with PKH26 and blood-derived CD3^+^ T cells labeled with CFSE mixtures were centrifuged at 200× *g* for 5 min. The pellet was incubated at 37 °C for different times, gently resuspended in 200 uL of PBS, and analyzed immediately for double-labeled cell conjugates using flow cytometry and Zeiss LSM710 confocal microscope (Jena, Oberkochen, Germany).

### 2.7. PEDV Infection and Transmission

Blood-derived CD3^+^ T cells were pulsed with PEDV (MOI = 0.1) for different times at 37 °C and washed extensively to remove unbound virus. PEDV-pulsed, blood-derived CD3^+^ T cells were co-cultured with autologous blood-derived CD3^+^ T cells labeled with PKH26 that had been maintained at 37 °C in RPMI 1640 medium. After the indicated time, PEDV-carrying blood-derived CD3^+^ T cells labeled with PKH26 were checked and analyzed using flow cytometry. The transmission of virus within MLN-derived CD3^+^ T cells was checked in the same manner as in blood-derived CD3^+^ T cells

### 2.8. Chemotaxis Assay

The migration of the blood-derived CD3^+^ T cells infected with PEDV or inactivated virus cells by ultraviolet (UV-PEDV) was assayed using a transwell system (Corning Incorporated Costar, Corning city, NY, USA) with a 5 μm pore size filter. Firstly, a medium containing different concentrations of CCL25 or MAdCAM-1 was added to the bottom chamber, the filters were dropped in the medium, and 5 × 10^5^ cells were immediately added. The chemokine MAdCAM-1 was expressed by prokaryotes and preserved in our lab. The transwell plates were incubated at 37 °C for 12 h and finally the cells in the lower compartment were collected and analyzed using a Cell Analysis Instruments system (JIMBIO PRO, Jiangsu, China)

### 2.9. Transfer Infection

Vero cells were seeded in 24-well plates and grown into monolayers before transfer infection. PEDV-carrying CD3^+^ T cells (MOI = 1) were co-cultured with Vero cells in direct contact. Negative controls, including uninfected T cells, and positive controls, which consisted of an identical number of PEDV viruses (MOI = 1), infected Vero cells. T cells and the medium of the Vero co-culture were collected at the indicated times. Vero cells were washed five times with PBS to eliminate non-absorbed virus and T cells, followed by culture in maintenance medium for indicated time periods.

### 2.10. Western Blot Analysis

Total protein from cells were lysed using lysis buffer. Tissues lysates were separated on 10% SDS–polyacrylamide gels and transferred to a polyvinylidene difluoride (PVDF) membrane (Biorad, Hercules, CA, USA). After blocking with 5% nonfat milk in Tris-buffered saline (TBS) buffer containing 0.05% Tween20, the membrane was incubated with a mouse mAb against PEDV N protein antibody, followed by HRP-conjugated secondary antibodies in the blocking reagent. After washing three times with TBST (Tris Buffered Saline Tween), immune reactive bands were analyzed by film exposure after enhanced chemiluminescence (ECL) (Millipore, Bedford, MA, USA) reaction.

### 2.11. Flow Cytometric Analysis

The cells were acquired from the different mucosa and stained with the indicated antibody. After surface staining, the cells were resuspended in fixation or permeabilization solution (BD Cytofix or Cytoperm Kit, respectively; BD Pharmingen) and stained with PEDV N protein antibody to detect intracellular PEDV. After three washes with PBS, the cells were phenotypically analyzed by FACS.

### 2.12. RT-qPCR Analysis

Total RNA was extracted from cells using RNAiso Plus kit (TaKaRa Bio, Dalian, China) following the manufacturer’s instructions. Fresh RNA (1 μg) was reverse-transcribed to cDNA with commercial oligo dT primers using Prime Script™ II 1st strand cDNA Synthesis Kit (TaKaRa Bio., Dalian, China). PCR was performed using a SYBR Green qPCR Kit (TaKaRa Bio., Dalian, China) in an Applied Biosystems 7500 Fast Real-Time PCR System (Life Technologies, Carlsbad, CA, USA). Gene expression was normalized to amplify glyceraldehyde 3-phosphate dehydrogenase (GAPDH). The data were analyzed using the 2^−ΔΔCT^ method.

### 2.13. Statistical Analysis

Results are expressed as the means ± SD and were analyzed with SPSS 17.0. One-way analysis of variance (ANOVA) was employed to determine significant differences among multiple groups, and t-tests were employed to determine the differences between two groups. Note: * *p* < 0.05, ** *p* < 0.01. Data were combined from at least three independent experiments unless stated otherwise.

## 3. Results

### 3.1. PEDV Bridles Blood-Derived CD3^+^ T Cells In Vitro and In Vivo

**Figure 1:**
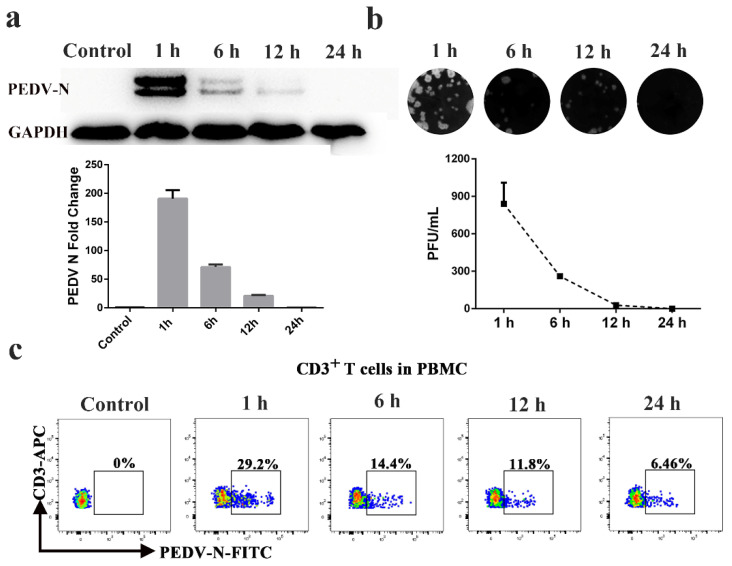
Blood-derived CD3^+^ T cells could be hijacked by PEDV in vitro. Blood-derived CD3^+^ T cells were infected with PEDV at different times. The viral loads in blood-derived CD3^+^ T cells were detected by Western blotting (**a**), plaque assay (**b**), and FACS (**c**). At least three independent experiments were performed. PBMC: Peripheral blood monoculear cell; FACS: Fluorescence Activated Cell Sorte.

**Figure 2:**
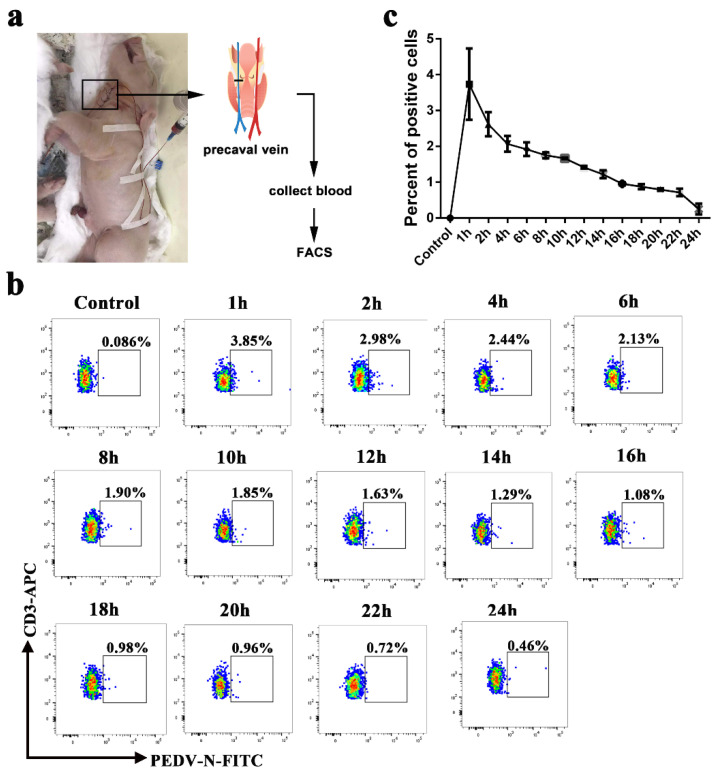
PEDV could be hijacked by blood-derived CD3^+^ T cells in vivo. (**a**). A schematic of vascular fistula in neonatal piglets. After PEDV intranasal inoculation, blood was collected at different times. The numbers of PEDV-loaded CD3^+^ T cells were analyzed using FACS (**b**) and statistics (**c**). FACS: Fluorescence Activated Cell Sorte.

CD3^+^ T cells derived from blood were stimulated by PHA and IL-2 and were inoculated with PEDV. At 1, 6, 12, and 24 h post infection (hpi), the CD3^+^ T cells and supernatant were collected for Western blot, FACS, and plaque assays to determine intracellular and extracellular virus titers. The Western blot and plaque assay results showed that the number of PEDV particles carried by a CD3^+^ T cell was the highest at 1 h after infection. With increases in infection time, the viral load gradually decreased (Figure 1a,b). FACS analysis presented similar results to the Western blot and plaque assays. The CD3^+^ T cells inoculated with PEDV reached a maximum of 29.2% at 1 hpi. The number of PEDV-positive CD3+ T cells gradually decreased from 14.4% to 6.46% over 6 to 24 hpi (Figure 1c). No significant differences in PEDV-positive CD3^+^ T cells were observed between untreated and treated CD3^+^ T cells using PHA and IL-2 (Figure S1). To determine the viral survival time in vivo in blood-derived CD3^+^ T cells, we performed venous fistula tests in neonatal piglets infected with PEDV by intranasal inoculation, followed by blood collection at different time points (Figure 2a). The FACS results showed that the percentage of PEDV-positive CD3^+^ T cells in the blood reached a maximum of 3.85% at 1 hpi and then decreased from 2.98% to 0.46% over 1 to 24 hpi (Figure 2b). The statistical results showed that viral load peaked at 1 h after intranasal injection and then gradually decreased within 24 h (Figure 2b,c). Based on these data, it appears that PEDV could be contained in the cytoplasm of blood-derived CD3^+^ T cells. These results indicate that blood-derived CD3^+^ T cells could carry PEDV and that the virus could survive in blood-derived CD3^+^ T cells for several hours.

### 3.2. The MOI Has an Impact on the Percentage of PEDV-Positive CD3^+^ T Cells

**Figure 3:**
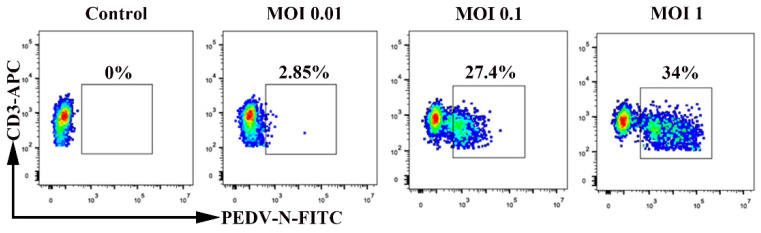
Multiplicity of infection affects the percentage of PEDV^+^ blood-derived CD3^+^ T cells. Blood-derived CD3^+^ T cells were inoculated at an MOI of 0.01, 0.1, or 1 with PEDV. Percentages of PEDV-N-positive CD3^+^ T cells were detected using FACS. FACS: Fluorescence Activated Cell Sorte; MOI: Multiplicity of infection.

We detected whether PEDV infection of blood-derived CD3^+^ T cells is dependent on the multiplicity of infection (MOI). Blood-derived CD3^+^ T cells were inoculated with PEDV at different MOI levels (0.01, 0.1, and 1). Increasing the MOI altered the number of PEDV-positive CD3^+^ T cells at 1 hpi. The increase in MOI from 0.01 to 0.1 increased the percentage of PEDV-positive CD3^+^ T cells. The percentage of PEDV-positive CD3^+^ T cells reached a steady level at an MOI of 0.1. Indeed, increasing the MOI from 0.1 to 1 did not significantly increase the percentage of PEDV-positive CD3^+^ T cells (Figure 3). These data suggest that PEDV infection of blood-derived CD3^+^ T cells is MOI-dependent.

### 3.3. PEDV Predominantly Bridles CD4^+^ T Cells

**Figure 4:**
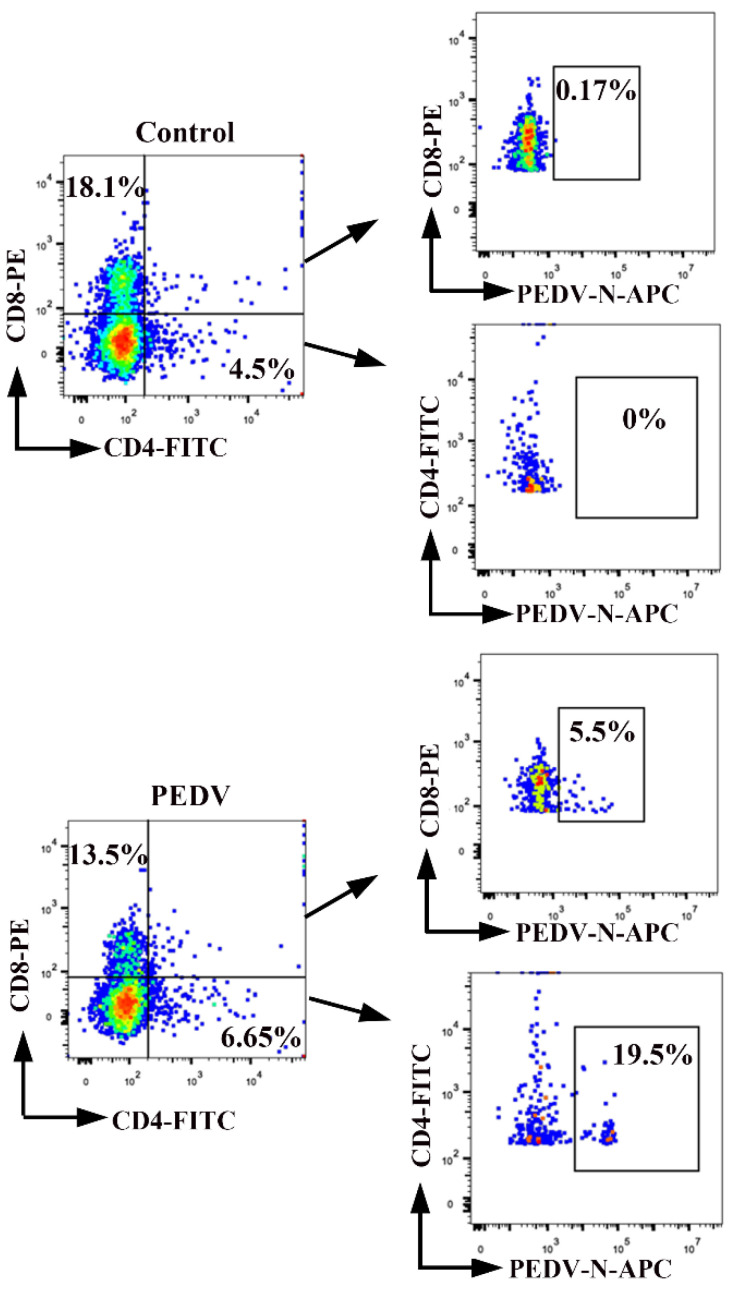
CD4^+^ and CD8^+^ subsets of T cells become infected with PEDV. PBMCs were inoculated at an MOI of 0.1 with PEDV. Percentages of PEDV-positive CD4^+^ T cells and PEDV positive CD8^+^ T cells were detected by FACS. The percentage of positive cells in each quadrant is shown. FACS: Fluorescence Activated Cell Sorte.

We determined which specific subsets of T cells are more susceptible to PEDV infection. Porcine PBMCs were isolated from the blood of neonatal piglets, followed by inoculation with PEDV (MO1 = 0.1). We detected PEDV-positive CD4^+^ T cells and PEDV-positive CD8^+^ T cells using FACS (Figure 4). Compared to non-infected control cells, the percentages of PEDV-positive CD4^+^ T cells and PEDV-positive CD8^+^ T cells were 19.5% and 5.5% at 1 hpi, respectively. The number of CD4^+^ T cells infected with PEDV was higher than that of CD8^+^ T cells infected with PEDV. In addition, a small percentage of CD8^+^ T cells showed PEDV signaling. These findings demonstrated that PEDV preferentially survives in CD4^+^ T cells over CD8^+^ T cells.

### 3.4. Cell-to-Cell Contact between Blood-Derived CD3^+^ T Cells Is Not Affected by PEDV Infection

**Figure 5:**
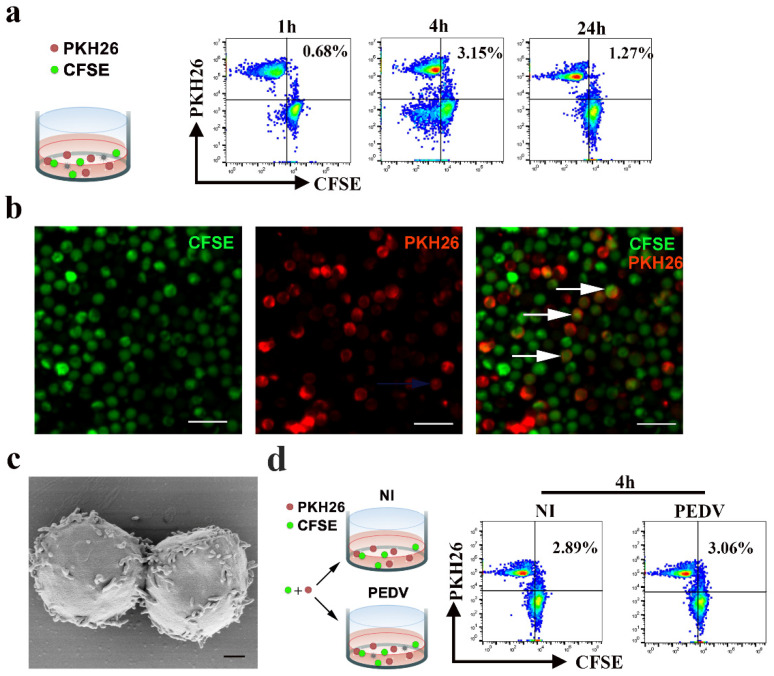
Cell-to-cell contact in CD3^+^ T cells of PBMC is not affected in PEDV infection. (**a**,**d**) For flow cytometry analysis, CD3^+^ T cells or CD3^+^ T cells infected PEDV were stained with the fluorescent probe PKH26 (red) and co-cultivated with target CFSE^+^ CD3^+^ T cells at a 1:1 ratio. The percentages of double-fluorescent cells among all cells, which correspond to cell-cell clustering or fusion, are depicted at the indicated time points. Data are representative of three independent experiments. NI, non-infected cells. (**b**) For confocal microscopy analysis, donors and targets were labeled with distinct membrane-associated fluorescent probes, which emittied in the red (PKH-26) or green (CFSE) wavelength. Formation of double-fluorescent cells in co-culture at a 1:1 ratio was detected by IF. The scale bar represents 20 μm. (**c**) The structures of lymphocyte-to-lymphocyte contacts were observed using a scanning electron microscope. The scale bar represents 1 μm. PKH26: Red Fluorescent Cell Linker Kits; CFSE: Carboxyfluorescein succinimidyl amino ester.

To detect whether cell-to-cell contact could occur among blood-derived CD3^+^ cells, we performed immunofluorescence (IF) and FACS experiments. Donor and target cells were labeled with distinct membrane-associated fluorescent probes, which emitted in red (PKH-26) or green (CFSE) wavelengths, respectively. Formation of dual-fluorescence cells in co-culture was detected using FACS and IF. The FACS results showed that the formation of dual-fluorescence cells was dependent on time and peaked at 4 hpi, reaching up to approximately 3.15% (Figure 5a). The IF results demonstrated that fusion or clustering events occurred among blood-derived CD3^+^ T cells (Figure 5b). Furthermore, the morphological characteristics of blood-derived CD3^+^ T cells were observed using a scanning electron microscope (SEM). The results of SEM visibly confirmed cell-to-cell contact, including numerous short microvilli randomly distributed over the entire surface of CD3^+^ T cells and close contact between blood-derived CD3^+^ T cells (Figure 5c). To determine whether cell-to-cell contact in blood-derived CD3^+^ T cells could be affected by PEDV, we quantified the formation of dual-fluorescence cells in co-culture with or without PEDV infection. Following FACS analysis, the fractions of dual-fluorescence cells after 4 h of co-culture were approximately 3.06% and 2.89% in the PEDV-infected and uninfected samples, respectively, suggesting that cell-to-cell contact was not affected by PEDV infection (Figure 5d).

### 3.5. PEDV Cell-to-Cell Transmission in MLN-Derived CD3^+^ T Cells

**Figure 6:**
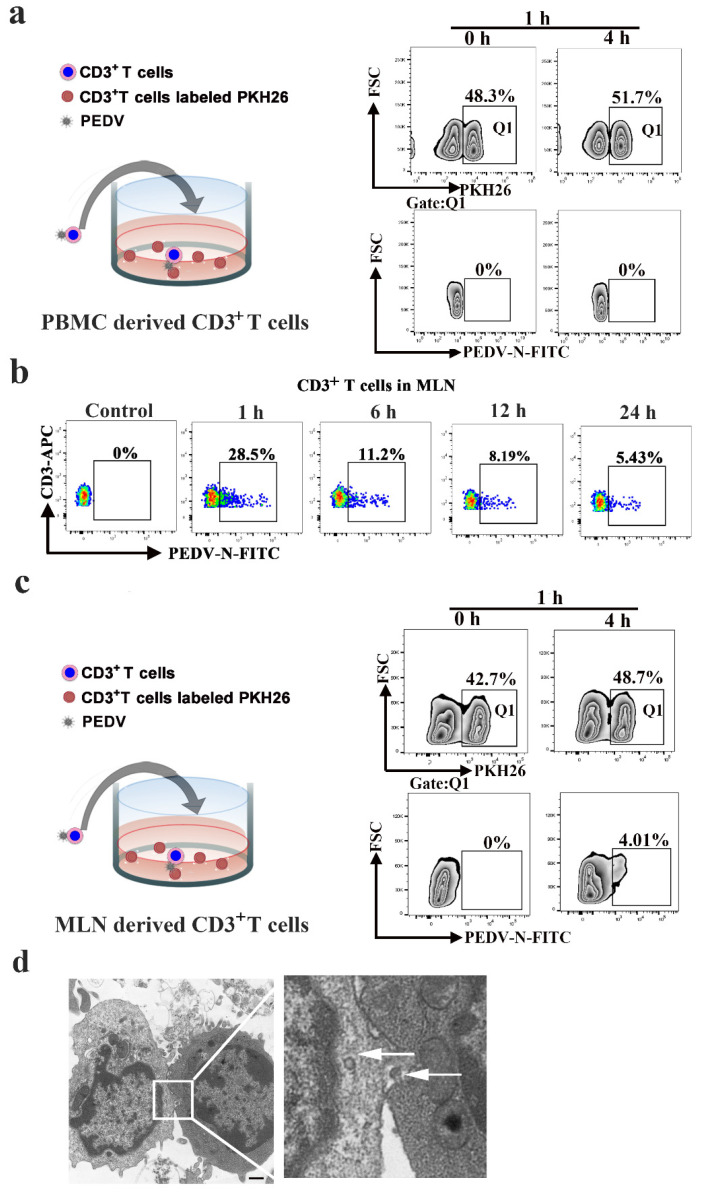
PEDV cell-to-cell transfer in CD3^+^ T cells of the lymph node. CD3^+^ T cells from PBMC (**a**) or the MLN (**c**) were infected with PEDV. These cells were then used as donors in the flow cytometry-based assay of viral transfer. Infected cells were co-cultivated with the indicated target PKH26 CD3^+^ T cells from PBMC or the MLN at a 1:1 ratio at the indicated time points. (**b**), CD3^+^ T cells from MLN were infected with PEDV at different times. The viral load values were detected using FACS. (**d**) The structures of lymphocyte-to-lymphocyte contacts were observed using transmission electron microscopy. In addition, we found complete virions at the site of cell-cell contact (arrowhead). The scale bar represents 1 μm. MLN: mesenteric lymph node. Q1:PKH26+ cells

Next, we studied whether PEDV could be transferred between blood-derived CD3^+^ T cells. The blood-derived CD3^+^ T cells used as donors were infected with PEDV (MOI = 0.1) for 1 h and the co-culture recipient cells were labeled with the membrane-associated fluorescent probe PKH-26 in co-culture. No significant viral transfer was detected when the target cells were separated from the donors in the co-culture system (Figure 6a). Mesenteric lymph node (MLN)-derived CD3^+^ T cells are different from blood-derived CD3^+^ T cells. Lymphocyte motility is slower in lymph nodes than in blood, facilitating productive contact between cells and leading to the formation of immunological or virological synapses [20,21]. First, the FACS results showed that the number of PEDV-positive CD3^+^ T cells from MLN reached a maximum value of 28.5% at 1 hpi and then dramatically decreased from 11.2% to 5.43% over 6 to 24 hpi, similar to that of blood-derived CD3^+^ T cells (Figure 6b). Then, we further investigated whether cell-to-cell transfer of PEDV would occur in MLN-derived CD3^+^ T cells. The FACS results showed that approximately 4.01% of target cells became positive for PEDV after 4 h of co-culture, indicating that PEDV-positive CD3^+^ T cells from MLN can efficiently transmit the virus to MLN-derived CD3^+^ T cells (Figure 6c). Moreover, transmission electron microscopy revealed the structures of cell-to-cell contact that provide the opportunity for viral transmission between MLN-derived CD3^+^ T cells, while the presence of complete virions at the site of cell-to-cell contact was also observed (Figure 6d).

### 3.6. Involvement of Integrin α4β7 in Blood-Derived CD3^+^ T Cells Carrying PEDV

**Figure 7:**
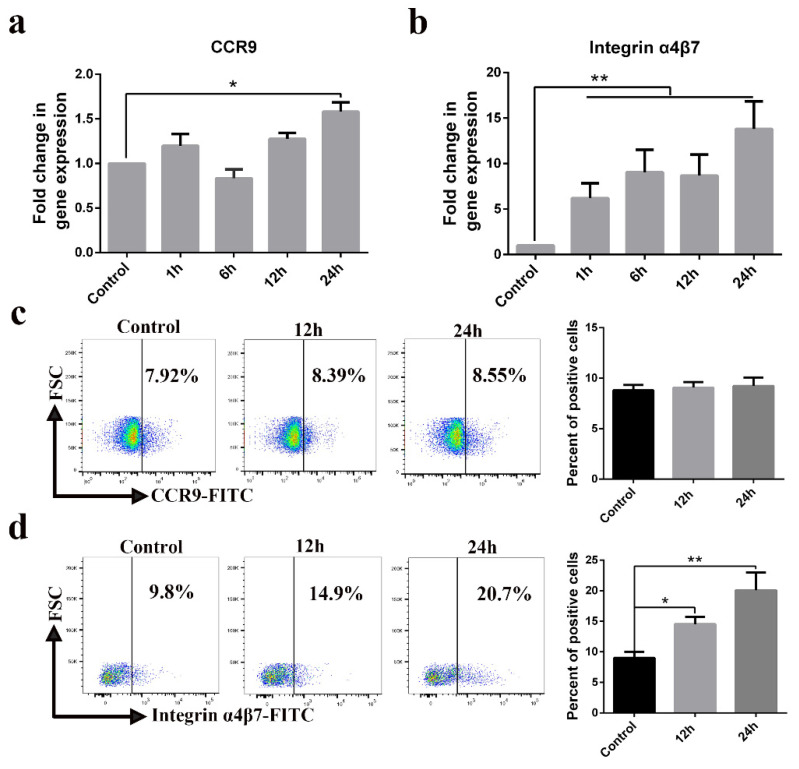
CD3^+^ T cell homing receptor integrin α4β7 is upregulated by PEDV infection. (**a**,**b**) The expression levels of CCR9 and integrin α4β7 were examined in CD3^+^ T cells infected with PEDV at indicated times using real-time RT-PCR. (**c**,**d**) The expression levels of CCR9 and integrin α4β7 were examined in CD3^+^ T cells infected with PEDV at the indicated time using FACS. Data are the means ± SDs. Statistical significance was assessed using one-way ANOVA. Note: * *p* < 0.05; ** *p* < 0.01. CCR9: C-C chemokine receptor.

**Figure 8:**
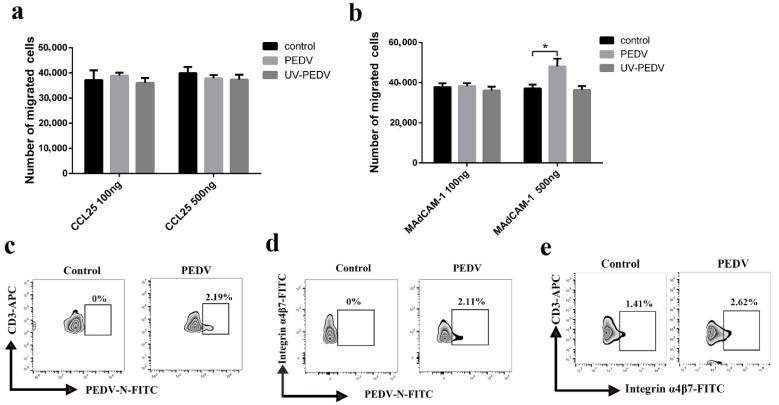
Expression of integrin α4β7 in CD3^+^ T cells promotes their migration. (**a**,**b**) CD3^+^ T cells or CD3^+^ T cells infected with PEDV or UV-PEDV (10^6^ cells/mL) were added into the upper chamber in the presence or absence of chemokines CCL25 and Madcam-1 overexpressed by prokaryotic expression in the lower chamber. After 12 h, counting of cells retrieved from the lower chamber were checked using a Cell Analysis Instruments system. Data are the means ± SDs. Statistical significance was assessed using one-way ANOVA. Note: * *p* < 0.05. (**c**–**e**) Neonatal piglets infected with PEDV underwent intranasal inoculation at 12 hpi and the numbers of PEDV^+^ CD3^+^ T cells, PEDV^+^ integrin α4β7^+^ T cells and integrin α4β7^+^ CD3^+^T cells were detected using FACS. UV: Ultraviolet.

The expression of CCR9 and integrin α4β7 on CD3^+^ T cells has been described as constituting a specific receptor that directs homing to the gut. We found high-level expression of CCR9 transcripts in PEDV-infected CD3^+^ T cells at 24 h (Figure 7a). High levels of integrin α4β7 transcripts were detected in PEDV-infected CD3^+^ T cells at a different time (Figure 7b). However, FACS analysis further validated that no difference in CCR9 expression was noted between PEDV-infected and uninfected CD3^+^ T cells (Figure 7c). PEDV infection increased the expression of integrin α4β7 on CD3^+^ T cells compared to expression on non-infected CD3^+^ T cells (Figure 7d). To study the involvement of CCR9 and integrin α4β7 in the chemotaxis of CD3^+^ T cells, we assessed their migration using a transwell system. The migration of CD3^+^ T cells infected with PEDV or UV-PEDV were assayed in transwell plates with 5 μm pore size filters. The CD3^+^ T cells infected with PEDV or UV-PEDV were added to the upper chamber and CCL25 or MAdCAM-1 was added to the lower chamber. The results showed no significant differences in the numbers of CD3^+^ T cells between the control, PEDV-, and UV-PEDV-infected cells in the presence of CCL25 (Figure 8a). However, PEDV-infected CD3^+^ T cells displayed marked increases in migration in the presence of 500 ng/mL MAdCAM-1 compare to control and UV-PEDV-infected cells (Figure 8b). To further evaluate whether PEDV-loaded CD3^+^ T cells expressing integrin α4β7 can reach the small intestine in vivo, neonatal piglets were infected with PEDV via intranasal inoculation. Using FACS analysis, 2.19% PEDV-positive CD3^+^ T cells and 2.11% PEDV-positive integrin α4β7^+^ T cells were detected at 12 hpi (Figure 8c,d). These data demonstrate that integrin α4β7, a homing receptor for CD3^+^ T cells derived from blood, plays an important role in PEDV infection in neonatal piglets via intranasal inoculation.

### 3.7. Blood-Derived CD3^+^ T Cells Carrying PEDV Cause Intestinal Infection via Cell-to-Cell Contact

**Figure 9:**
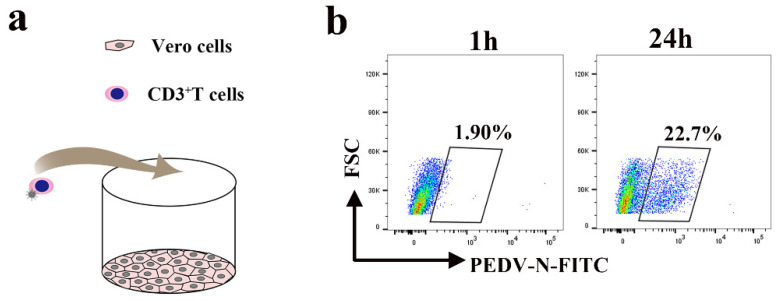
PEDV-loaded CD3^+^ T cells mediate transfer infection. (**a**) The model of the co-culture assay with blood-derived CD3^+^ T cells infected with PEDV and Vero. (**b**) Blood-derived CD3^+^ T cells infected with PEDV and co-cultivated with Vero cells at the indicated time points. The CD3^+^ T cells were washed, then PEDV^+^ Vero cells were detected using FACS.

Lymphocytes typically located beneath the intestinal epithelial cells may also be interspersed between the intestinal epithelial cells of neonatal piglets. Close contact between lymphocytes and intestinal epithelial cells facilitates transmission of the virus. To evaluate whether virus-loaded CD3^+^ T cells can cause intestinal infections, we performed a co-culture assay (Figure 9a). The co-culture assay was performed with blood-derived CD3^+^ T cells infected with the virus for 1 h and epithelial cells (Vero cells). After 24 hpi, up to 21.8% of Vero cells were positive for PEDV, as assessed by FACS (Figure 9b). Removal of the virus in the supernatant at 1 hpi eliminated the possibility of residual PEDV outside of CD3^+^ T cells in the medium (Figure S2).

## 4. Discussion

PED, which causes significant morbidity and mortality in neonatal piglets, has caused global concern [6]. Most existing studies of PED have focused on its pathogenesis via the fecal-oral route [4]; however, fewer studies have been conducted to delineate the underlying mechanisms of the fecal–nasal route. The nasal cavity is a route of entry for intestinal-infection-causing PEDV in piglets, while the migration of CD3^+^ T cells carrying PEDV plays an important role in PEDV infection through this route [7,8]. We demonstrated that PEDV could somehow be contained in the cytoplasm of CD3^+^ T cells. These results are in accordance with previous reports of PEDV [7]. However, how PEDV invades or binds to CD3^+^ T cells remains unclear. To address this question, more research is warranted.

Viral spread via cell-to-cell contact is a common strategy in viral infection. Viral transmission between immune cells was first discovered in varicella zoster virus (VZV) infection, in which VZV-infected DCs could transfer infectious virus to autologous human T cells [22]. This phenomenon was later observed in the pathogenesis of HIV, where stable adhesive contacts formed between immune cells facilitated viral transfer from infected cells to uninfected cells [23]. HIV can be transmitted between DCs and lymphocytes, as well as within lymphocytes [24,25]. Viral transmission from one immune cell to another primarily involves two methods of transmission—from DCs to lymphocytes or between lymphocytes. Some studies have shown that viruses can be preferentially transmitted via contact between infected and uninfected T cells [23,26,27]. Moreover, the magnitude of stable, adhesive-contact-mediated viral antigen transfer is significantly greater than that of viral antigen transfer mediated by high levels of cell-free virus [12]. This strategy is also exploited by PEDV. We demonstrated that PEDV can be transmitted from PEDV-positive CD3^+^ T cells to other CD3^+^ T cells within lymph nodes, but not in the blood-derived CD3^+^ T cells. One plausible explanation is the different cell surface phenotypes of T lymphocytes derived from peripheral blood and the lymph nodes. CD3^+^ T cells carrying PEDV are able to enter the lymphatic and blood circulatory systems. PEDV not only uses CD3^+^ T cells as transmission vehicles but also to amplify the infection by transmitting virus from one infected CD3^+^ T cell to multiple uninfected CD3^+^ T cells.

The T cell functions that aid viral entry into the intestinal mucosa constitute a complex process that is regulated by selective expression of intestinal homing receptors on the T cells’ surfaces, as well as of corresponding ligands within the intestinal mucosa. The intestinal homing receptor components α4β7 and CCR9 are selectively induced in T cells primed in the small intestine. Interactions between integrin α4β7 in circulating effector T cells and its ligand MAdCAM-1 in intestinal microvascular endothelial cells are important for efficient T cell extravasation into the small intestine [28]. Similarly, the CCR9 ligand, CCL25, which is selectively expressed in the small intestine, is also important for efficient T cell entry into the small intestine [29]. Furthermore, interactions between CCR9 and its ligand CCL25 promote lymphocytic migration across the surface of the epithelial cell basal membrane, as well as their transformation into intraepithelial lymphocytes (IELs) [30,31]. Several studies have shown that integrin α4β7 is efficiently incorporated into the envelope of HIV-1 virions in order to promote their trafficking to the gut via interactions with MAdCAM-1 [19]. MAdCAM-1 is highly expressed in high endothelial venules within the intestinal mucosal surface. Consistent with other findings, we found that PEDV infections can upregulate the expression of integrin α4β7 in CD3^+^ T cells. One possible explanation is that the interaction between PEDV and integrin α4β7 in CD3^+^ T cells might stimulate the expression of integrin α4β7. Another possibility is that PEDV might generate or combine with other cytokines to promote the expression of the gut-homing molecule integrin α4β7 in CD3^+^ T cells. Elevated expression of gut-homing integrin α4β7 in T cells in PEDV infections promotes their trafficking into the gut via interaction with MAdCAM-1. However, the reason for the lack of a difference in CCR9 expression between PEDV-infected and uninfected CD3^+^ T cells remain unclear.

T cells can serve as important reservoirs of infection. Lymphocytes readily transmit the virus to target cells (susceptible cells) at the site of disease. This phenomenon has been confirmed in a variety of viruses. For example, lymphocytes that carry HIV can transmit the virus to astrocytes in brain tissue, across the blood–brain barrier [32]. Infected lymphatic cells can carry measles virus (MV) to the basolateral surface of epithelial cells, supporting MV shedding into the airway lumen, and thereby facilitating contagion [33,34]. B cells carrying the Epstein–Barr virus also transmit the virus to epithelial cells [18,35]. In line with our a priori hypotheses, this phenomenon was further confirmed in PEDV. We found that CD3^+^ T cells carrying PEDV directly transmit the virus to Vero cells via cell-to-cell contact.

To our knowledge, this study is the first to examine the pathogenesis mechanism of intestinal mucosa infection caused by enteroviral entry via nasal invasion and circulatory diffusion. PEDV-hijacked T cells are migratory vehicles for viral dissemination in neonatal piglets. Our results reveal the mechanism underlying the pathogenesis of the intestinal mucosa infection caused by PEDV in neonatal piglets and provides a theoretical basis for the prevention and control of widespread PED.

## Data Availability

Not applicable.

## Supplementary Materials

The following are available online at https://www.mdpi.com/1999-4915/13/3/469/s1, Figure S1: PHA and IL-2-mediated stimulation of blood-derived CD3^+^ T cells does not enhance susceptibility to PEDV infection, Figure S2: The virus titer was detected by plaque assay.

## Abbreviations

PEDV	Porcine epidemic diarrhea virus
PED	Porcine epidemic diarrhea
DCs	Dendritic cells
MOI	Multiplicity of infection
IHC	Immunohistochemistry
IF	Immunofluorescence
TEDs	Transepithelial dendrites
CCR9	C-C chemokine receptor 9
CCL25	C-C chemokine ligand
MAdCAM-1	Mucosal addressin cell adhesion molecule 1
HIV-1	Human immunodeficiency virus type-1
PVDF	Polyvinylidene difluoride
TBS	Tris-buffered saline
hpi	Hours post infection
SEM	Scanning electron microscope
MLN	Mesenteric lymph nodes
VZV	Varicella zoster virus
MV	Measles virus
