# Supplementary material for: The Mechanism of PEDV-Carrying CD3+ T Cells Migrate into the Intestinal Mucosa of Neonatal Piglets
Source: Viruses. 2021 Mar 12;13(3):469. doi: 10.3390/v13030469 (PMC8000367; doi:10.3390/v13030469)
Supplement: Supplementary file 1 [file viruses-13-00469-s001.pdf]

Supplementary Information

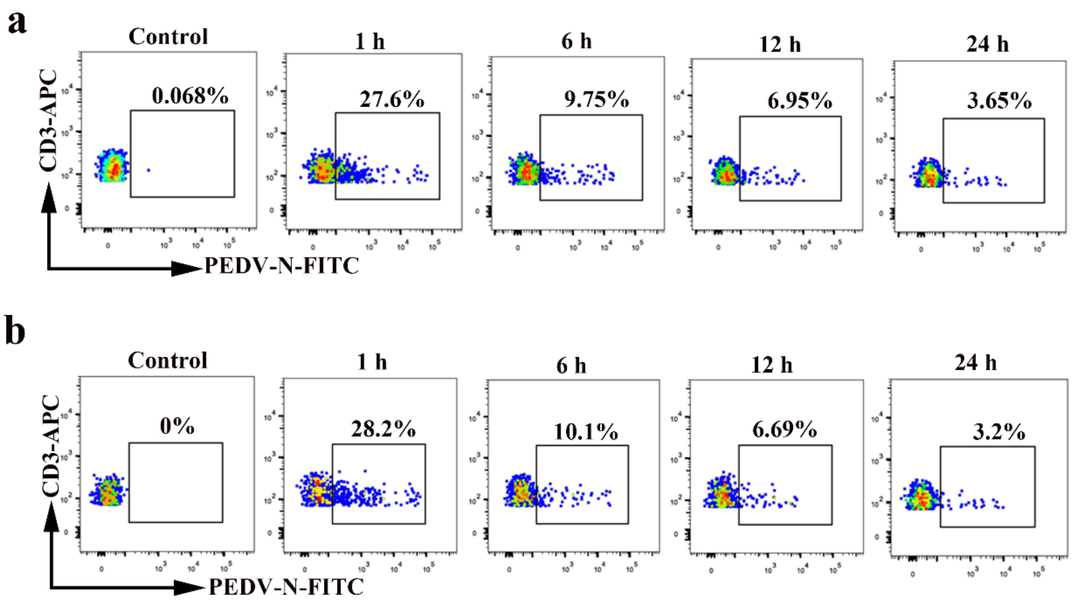

Fig.1 PHA and IL-2-mediated stimulation of blood-derived CD3^{+} T cells does not enhance susceptibility to PEDV infection. Primary blood-derived CD3^{+} T cells (a)or blood-derived CD3^{+} T cells stimulated with PHA and IL-2 (b)were inoculated at an MOI of 0.1with PEDV. Percentages of PEDV positive CD3^{+}T cells were detected by FACS.

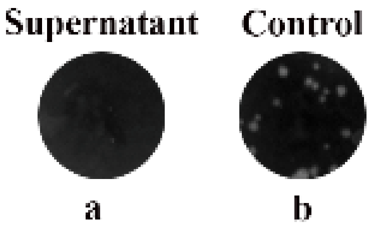

Fig.2 The virus titer was detected by plaque assay

a. Virus in the supernatant of blood-derived CD3^{+}T cells infected PEDV at 1 h. b. PEDV as positive control
